# Using a Mobile App to Promote Smoking Cessation in Hospitalized Patients

**DOI:** 10.2196/mhealth.5149

**Published:** 2016-05-06

**Authors:** Joseph Finkelstein, Eun Me Cha

**Affiliations:** ^1^Columbia UniversityDepartment of Biomedical InformaticsNew York, NYUnited States; ^2^Michael & Susan Dell Center for Healthy LivingThe University of Texas School of Public HealthAustin, TXUnited States

**Keywords:** mobile apps, patient engagement, hospital, smoking cessation, health literacy

## Abstract

**Background:**

The potential of interactive health education for preventive health applications has been widely demonstrated. However, use of mobile apps to promote smoking cessation in hospitalized patients has not been systematically assessed.

**Objective:**

This study was conducted to assess the feasibility of using a mobile app for the hazards of smoking education delivered via touch screen tablets to hospitalized smokers.

**Methods:**

Fifty-five consecutive hospitalized smokers were recruited. Patient sociodemographics and smoking history was collected at baseline. The impact of the mobile app was assessed by measuring cognitive and behavioral factors shown to promote smoking cessation before and after the mobile app use including hazards of smoking knowledge score (KS), smoking attitudes, and stages of change.

**Results:**

After the mobile app use, mean KS increased from 27(3) to 31(3) (*P*<0.0001). Proportion of patients who felt they “cannot quit smoking” reduced from 36% (20/55) to 18% (10/55) (*P*<0.03). Overall, 13% (7/55) of patients moved toward a more advanced stage of change with the proportion of patients in the preparation stage increased from 40% (22/55) to 51% (28/55). Multivariate regression analysis demonstrated that knowledge gains and mobile app acceptance did not depend on age, gender, race, computer skills, income, or education level. The main factors affecting knowledge gain were initial knowledge level (*P*<0.02), employment status (*P*<0.05), and high app acceptance (*P*<0.01). Knowledge gain was the main predictor of more favorable attitudes toward the mobile app (odds ratio (OR)=4.8; 95% confidence interval (CI) (1.1, 20.0)). Attitudinal surveys and qualitative interviews identified high acceptance of the mobile app by hospitalized smokers. Over 92% (51/55) of the study participants recommended the app for use by other hospitalized smokers and 98% (54/55) of the patients were willing to use such an app in the future.

**Conclusions:**

Our results suggest that a mobile app promoting smoking cessation is well accepted by hospitalized smokers. The app can be used for interactive patient education and counseling during hospital stays. Development and evaluation of mobile apps engaging patients in their care during hospital stays is warranted.

## Introduction

Smoking remains the most common cause of preventable mortality and morbidity in the United States [1] and the leading risk factor for global disease burden [2]. Tobacco consumption resulted in 435,000 deaths in the United States (18% of total US deaths) in 2000 [3]. Even after adjusting for multiple sociodemographic, behavioral, and health-related risk factors, overall estimate of deaths attributable to smoking in the United States was shown to be approximately 400,000 per year [4]. Although significant progress has been achieved in reducing smoking rates, one in five adults in the United States is a current smoker, with smoking prevalence remarkably higher among adults with lower educational attainment [5].

National surveys suggest continued need for patient education about smoking hazards [5]. While information on increased risk for cardiovascular disease and lung cancer has been widely publicized, many smokers still lack knowledge about cigarette smoking’s relationship to other types of cancer, reproductive health problems, premature disability, and reduced quality of life [6]. Gender, age, racial, and socioeconomic disparities in knowledge and beliefs about smoking have been demonstrated, with males, older adults, non-Hispanic blacks, Hispanics, and those with lower incomes being significantly more likely to believe in myths such as reversal of smoking effects by exercise and vitamin intake [5]. While only 5% of graduate students smoke, 27% of adults with less than a high school diploma and 41% of those with a General Educational Development certificate are current smokers [1]. Recent studies demonstrated that lower knowledge of hazards of smoking is associated with higher tobacco use and higher tobacco-related morbidity and mortality [5]. These studies underscored the need for continued development and delivery of effective means to address disparities in tobacco-related knowledge.

Hospitalization offers an opportunity to provide smokers with advice, education, and counseling. Acute illness may increase a patient’s motivation and has been described as a teachable moment that providers should not miss [7-9]. Hospital-initiated interventions for smoking cessation have been demonstrated to increase long-term quit rates [10] and even brief advice has been demonstrated to be of value when offered by providers [9,11]. However, systematic delivery of in-hospital, patient-tailored education and counseling on smoking continues to be far from routine [9,12] and only 18% of smokers overall abstain from smoking post hospitalization [13]. Language barriers, health literacy levels, lack of provider time, and limited resources are all factors that may contribute to this problem [14]. Multiple studies provided evidence that interactive health education programs can be instrumental in addressing these barriers [15-16].

Interactive health education programs delivered as mobile apps by touch screen tablets or smartphones have been demonstrated to increase knowledge, skills, and self-efficacy levels among patients with asthma, cancer, diabetes, congestive heart failure, chronic obstructive lung disease, and other conditions [17-18]. Such mobile apps, generally termed mHealth applications, may offer versatility and personalization, and may incorporate features that promote ease of use, use spoken and written language, use multiple languages, can be scripted at a level that addresses the needs of low literacy and numeracy learners, and may be viewed as often as needed by a patient [17-19]. Mobile apps supporting interactive education have the potential to greatly increase interest, because the learner actively participates in the learning process [20]. Limited computer experience and low-health literacy, which are more prevalent in individuals from low-socioeconomic strata, does not appear to impact their ability to use interactive health education programs effectively [21].

Recent studies have supported the use of mobile apps for smoking cessation [19-20,22,23]. Mobile apps for smoking cessation have been successfully implemented in a variety of settings and populations using multiple approaches and theoretical frameworks [24-26]. Comprehensive reviews of popular mobile apps for smoking cessation concluded that these apps can serve as powerful tools for smoking cessation in the future [27,28]. The review recommended that mobile apps for smoking cessation are developed in compliance with evidence-based principles and undergo rigorous evaluations [27,28]. A recent survey reported that the majority of health care providers embrace use of mobile apps for helping their patients quit smoking [29]. Despite wide introduction of mobile apps for health promotion to the general public, the use of mobile apps aimed at promoting smoking cessation in hospitalized patients has not been studied yet systematically [30].

In a previous study, we assessed the feasibility of promoting smoking cessation in the outpatient setting using an interactive health education program [31]. After completion of the “Hazards of Smoking Educational Program” delivered via a touch screen tablet, low-literacy patients with minimal computer skills exhibited a significant increase in knowledge levels about hazards of smoking and reported ease of system use [31]. In the current study, we sought to elucidate feasibility and potential impact of a mobile app to educate smokers in the hospital setting. Our objectives were to (1) describe the sociobehavioral characteristics of hospitalized smokers to inform future mobile app development for this population, (2) assess impact of the mobile app on smokers’ knowledge levels and behavioral factors associated with smoking cessation, (3) identify factors affecting users’ knowledge gain, (4) identify factors affecting patient acceptance of the mobile app in a hospital setting.

## Methods

### Participants

We conducted a prospective study of active smokers consecutively admitted to two medicine units at two large urban academic teaching hospitals. Fifty-five consecutive adults aged 18 years or older hospitalized for any reason and who were active smokers and agreed to participate, were enrolled into the study. The study protocol was approved by the institutional review board.

### Mobile App

The mobile app employed in this study was based on the COmputer-assisted EDucation system (CO-ED), which was previously described [31-35]. Briefly, CO-ED is designed to deliver interactive health education via multiple health communication channels [32,33] and was successfully used for patient education on a variety of health-related topics including asthma [34], diabetes [35], hypertension [36], depression [37], multiple sclerosis [38], ileostomy [39], smoking cessation [40], and Tai Chi [41]. The system consists of three components: knowledge repository containing educational content in a relational database format, teaching engine delivering educational content in concordance with major constructs of adult learning theories, and user interface supporting content delivery via multiple platforms including desktops, touch screen tablets, smartphones, gaming appliances (Wii and Xbox), and interactive voice response [33]. The CO-ED system is guided by principles of adult learning [42] and instructional technology foundations [43] using constructs from the Information Processing Theory [44], Constructivist Theory [45], Cognitive Flexibility Theory [46], Subsumption Theory [47], Drive Reduction Theory [48], and Cognitive Load Theory [49]. Applications of specific constructs from these theories in the CO-ED system were described in detail previously [33,50].

Previous studies emphasized importance of usability factors for successful acceptance of computer-assisted education especially in older adults and individuals with limited computer experience [35-39,51]. In this study, hospitalized patients were provided a touch screen tablet with a mobile app delivering computer-assisted education on the hazards of smoking. A touch screen tablet was chosen because of larger form factor as compared with a smartphone and because some of the hospitalized patients didn't use smartphones. The educational curriculum on hazards of smoking is written at the 5^th^grade level and based on a previously developed curriculum that has been adapted for use in the CO-ED system [31]. Brief educational statements about the effects of smoking and the feasibility of quitting are presented, each followed by a multiple choice question about the material. A voice-over option is available for people with functional illiteracy. The curriculum was comprised of five sections: (1) Is cigarette smoking dangerous? (2) How does smoking cause cancer? (3) How does smoking cause heart disease, stroke, and blocked arteries? (4) How does smoking cause lung disease? (5) Common questions about smoking. Overall, the curriculum reflected key content areas promulgated by recent recommendations including the dangers and effects of cigarette smoking as well as information on how to quit [1,51].

### Intervention

As this project was undertaken in preparation to wide introduction of tablet-based education for hospitalized patients, patient enrollment procedures were made as close to routine hospital workflow as possible. Hospital unit census was reviewed by a unit nurse on a daily basis to identify hospitalized smokers. Eligible participants were approached by a unit nurse and asked if they are interested to take part in the study. Interested patients were consented by study's research assistant. After obtaining informed consent, the research assistant provided each patient with a set of questionnaires to fill out and then provided a touch screen tablet with which the patient accessed a self-paced interactive education app on hazards of smoking. Patients spent up to 45 minutes using the mobile app independently without research assistant or nurse present. At the end of the 45-minute period, patients were approached by a research assistant again and asked to fill out a post-education survey. A 15 to 20 minute semistructured interview was also conducted at the end of each session so that patients could offer feedback on the feasibility of using the mobile app and ways to improve it.

### Data Collection and Study Instruments

Prior to system use, participants completed a set of questionnaires with questions about demographics, prior experience with mobile devices, and the Fagerstrom Test for Nicotine Dependence (FTND) [52]. Though the primary objective of our intervention was increase in hazards of smoking knowledge, we also were interested in elucidating the extent of impact of potential knowledge change on major behavioral constructs that are frequently used to explain smoking cessation behavior. Thus, cognitive and behavioral factors known to be associated with smoking cessation were collected to ascertain impact of the mobile app. A Knowledge Score (KS) questionnaire, Process of Smoking Cessation survey (to assess stages of change per Transtheoretical Model (TTM)), Smoking Self-Efficacy questionnaire, and Decisional Balance Scale were completed by participants pre- and post-mobile app use. To assess each participant’s experience with and opinions about the mobile app, an attitudinal survey and semistructured interview were administered to each of the study participants after using the mHealth education app.

The KS questionnaire is composed of 34 true or false questions asking basic information about smoking and negative impact of smoking on health. Examples of questions include: “Smoking during pregnancy is linked with a greater chance of miscarriage” and “Smoking only affects the lungs.” An identical questionnaire was completed before and after using the system. A perfect KS on the test is 34. The reliability of the KS scale assessed by the Cronbach’s alpha in our studies including the current one has been 0.75 and higher [31,50].

The Process of Smoking Cessation survey measures four different stages of quitting based on the TTM of change [53]. In the precontemplation stage, the smoker is not seriously thinking about changing the smoking behavior. In the contemplation stage, the smoker is more aware of the health consequences of smoking and starts thinking about quitting. In the preparation stage, the smoker has made a decision to stop smoking and is starting to take small steps toward cessation. In the action stage, a smoker believes that s/he has the ability to quit smoking and is actively involved in changing their smoking behavior. In the maintenance stage a person has changed and is now trying to maintain the change [53,54].

The Smoking Self-Efficacy questionnaire [55] is composed of 20 questions assessing confidence in ability to avoid smoking. It is divided into three sections reflecting three relapse situations: positive affect/social situations, negative affect situations, and habitual/craving situations with the highest section score of 30, 30, and 25, respectively. Three sections inquire about social factors, negative emotional states, and physiological factors like cravings and urges, which might trigger smoking. Higher scores are seen among those smokers who are more tempted to smoke.

The Decisional Balance Scale [56] is designed to assess and predict smoking behavior. It consists of 20 scales and is divided into two sections with 10 questions each. The Pros scale contains items representing the pleasure, tension reduction, self-image, and habit factors identified as the basic reason for smoking. The Cons scale items represent the health examples, aesthetics, and mastery considerations associated with motives for quitting. The score ranges for both Pros and Cons between 10 and 50. The comparison of Pros and Cons provides an insight on individuals’ status regarding their decision to continue or discontinue smoking [56].

The Attitudinal Survey assesses patients’ acceptance of the mobile computer-assisted education system and their perceptions of usability, content clarity, and usefulness of the system. The survey, which is composed of 18 items, was developed based on a literature review and critical feedback from experts in the field. The maximum survey score is 72. This survey has been used and validated in our previous studies [31-34].

The semistructured interview conducted at the end of the study explored participant opinions on educational content and the app interface. Participants were also asked to highlight mobile app benefits and drawbacks and to suggest areas for improvement. A qualitative thematic analysis was conducted using framework approach [57].

### Data Analysis

Data were analyzed using SAS statistical software version 9.2 [58]. We calculated difference in knowledge test scores for pre- and post-system use for each participant along with a composite score for the attitudinal survey. To check for statistical significance we used paired *t* -tests and two sample *t* -tests, as applicable, for continuous variables. We used two-tailed tests with a .05 significance level. To assess impact of variables such as race, age, and computer skills on knowledge gain from the mobile app, a multivariate linear regression model was used with difference in KS (DKS) before and after the mobile app use as dependent variable and age, race, gender, computer skills, educational level, and baseline KS as covariates. Pre/post proportions were compared using two-sided chi-square test. To check for the potential impact of various variables on participants’ attitudes toward using the mobile app, a multivariate linear regression model was used with the composite score on the attitudinal survey as the dependent variable and age, race, gender, computer skills, educational level, and KS difference as covariates. Mean (M) and standard deviation (SD) for continuous variables are reported in the following notation: M(SD).

Qualitative interviews were transcribed verbatim. The interview transcripts were independently analyzed by two researchers. A coding scheme was used to reflect themes that emerged from the data following a framework approach in analysis of qualitative data [57,59]. A comprehensive search was conducted for expressions indicating information types and the context of use, and possible problems when looking for or using information [57]. Adapted from a taxonomy that resulted from similar research [60], the information types were coded into (1) interface-specific, (2) content-specific, and (3) process-specific. Differences in coding by the two independent researchers were reviewed and reconciled until agreement was reached.

## Results

### Sociobehavioral Characteristics of Hospitalized Smokers

Participants’ demographic and socioeconomic status is detailed in Table 1. Overall, 55 eligible patients consented and participated in this study which constituted three-quarters of initially approached adult hospitalized smokers. Main reasons for refusal to consent were being too tired, too sick, or being distracted by upcoming tests or procedures. The mean age of study participants was 46.9(11.36)-years old ranging from 18 to 69 years.

**Table 1:** Participant sociodemographic characteristics.

Sociodemographic characteristics		N=55	%
**Gender**			
	Male	30	55
	Female	25	45
**Current relationship status**			
	Married/Common-law/Partner	15	27
	Single/separated/divorced/widowed	40	73
**Education**			
	<12 years	18	33
	12 years	24	44
	>12 years	13	24
**What is your level of computer skills?**			
	None/basic	31	56
	Good/advanced	24	44
**Current employment status**			
	Employed	12	22
	Unemployed	43	78
**What is your overall household income for the last year?**			
	<20K	19	35
	20K-40K	12	22
	>40K	9	16
	Prefer not to disclose	15	27
**Race**			
	Caucasian	23	42
	African American	30	55
	Other	2	4

Women constituted 45.5% (25/55) of enrolled smokers, 55% (30/55) were African Americans, and 42% (23/55) were Caucasians. Approximately 20% (11/55) of the study participants had a full-time employment, 33% (18/55) did not have high school diploma, and 44% (24/55) completed high school. Thirty-five percent (19/55) reported a low income household (<US$20,000 annual income per household). Fifty-six percent (31/55) had only a basic level of computer skills or no computer skills, and 53% (29/55) reported using a computer no more than once per week. Based on the patient chart review, 76% (42/55) of the study subjects were hospitalized for emergency treatment, 14% (8/55) for complex diagnostic procedures, and the rest for surgery or other treatment. Alcohol and drug abuse was the most frequent comorbid condition (20/55, 36%), followed by depression or other emotional problems (19/55, 34%), hypertension (15/55, 27%), heart disease and stroke (12/55, 22%), chronic obstructive pulmonary disease and asthma (12/55, 22%), diabetes (6/55, 11%), and cancer (5/55, 9%).

Smoking history of the study participants is summarized in Table 2. The subjects smoked an average of 13.6(9.1) cigarettes per day for 26.6(13.7) years. The participants started smoking at 18 years of age on average. Twenty-seven percent (15/55) of participants had never attempted to stop smoking, while 65% (36/55) had made at least one successful attempt that lasted for a full month or longer. Forty percent (22/55) of the study participants reported that they were ready to quit within 30 days and have made at least one 24-hour quit attempt during the past year. Another 43% (24/55) were thinking of quitting within the next 6 months. According to the findings from the FTND, half of the study participants typically smoked a cigarette within 5 minutes of waking, and 67% (37/55) answered that the first cigarette was the one they most hate to give up during the day. The mean FTND score for the participants was 4.7(2.8). FTND score of 5 or more indicates significant dependence, while a score of 4 or less shows a low to moderate degree of dependence. Fifty-six percent of study subjects (31/55) had FTND score above 5 demonstrating significant smoking dependence.

**Table 2:** Participant smoking history.

Smoking history characteristics (Mean (SD))		N=55	%
How many cigarettes a day do you smoke in average?		13.6 (9.1)	
How many days have you been already in the hospital?		3.3 (2.1)	
**How many days have you been smoking while in the hospital?**			
	0 (ie, no smoking in the hospital)	44	80
	1-2	8	15
	3	3	5
How many persons in your household smoke?		2.0 (1.5)	
How many years have you smoked cigarettes regularly?		26.6 (13.7)	
How old were you when you started smoking cigarettes?		18.2 (10.4)	
How many times have you SERIOUSLY tried to stop smoking?		2.4 (2.5)	
**What is longest number of months you have not smoked, not even a puff?**			
	Never stopped smoking	15	27
	Less than a month	4	7
	At least one month	36	65
**In the past year, have you stopped smoking cigarettes for at least one day (24-hours)?**			
	No	10	18
	Yes	45	82
**How seriously would you like to give up smoking altogether?**			
	Not at all	4	7
	Not very seriously	6	11
	Fairly seriously	14	25
	Very seriously	31	56

### Impact of the Mobile App on Smokers’ Knowledge Levels and Behavioral Factors

Table 3 summarizes effect of the mobile app on cognitive and behavioral factors associated with smoking cessation behavior. The use of the mobile app resulted in increase of hazards of smoking knowledge assessed by the KS questionnaire from 27.4 (2.6) to 30.5(3.1). This increase was statistically significant (paired *t* -test; *P*<0.0001). Based on the subjects' answers to the baseline KS questionnaire, participants already had good knowledge at baseline about addictive properties of tobacco and the increased risk for stroke, heart disease, lung cancer, and other respiratory tract cancers among smokers. They, however, lacked knowledge about other systemic effects of tobacco use such as increased risk for leukemia, colon cancer, and cervical cancer.

**Table 3:** Smoking knowledge and attitudes before and after the mobile app use.

Cognitive and behavioral factors of smoking cessation		Pretest	Posttest	*t* -test
	Knowledge Score Questionnaire (mean±(SD^a^))	27.4 (2.6)	30.5 (3.1)	0.0001^b^
**Attitudes toward smoking**		**%**	**%**	**χ**^**2**^ ***( P )***
	I cannot quit smoking	36	18	4.6 (0.03)^b^
	I have no desire to quit smoking	18	13	0.6 (0.43)
	I would lose a lot in my life if I quit smoking	15	11	0.3 (0.57)
	Health risks of smoking are exaggerated	20	11	1.7 (0.19)
	If I continue to smoke, my risk of dying from smoking-related disease is significantly higher comparing with an average nonsmoker	89	95	1.1 (0.30)
**Self-efficacy/temptation factors**^c^		**Mean±(SD)**	**Mean±(SD)**	***t*** **-test**
	Positive Affect/Social Situations	21.3 (5.9)	20.8 (6.3)	0.64
	Negative Affect Situations	24.5 (5.0)	23.4 (5.7)	0.26
	Habitual/Craving Situations	16.3 (4.9)	15.9 (5.2)	0.69

^a^SD: standard deviation.

^b^Pre/post difference is statistically significant.

^c^Higher the score, more tempted to smoke.

The mean baseline KS was significantly lower among African Americans than among Caucasians (African American KS = 26.7(2.8), range=19.0-31.0; Caucasian KS = 28.4(2.1), range=25.0-33.0; *P* value=.02). Both African American and Caucasian participants had higher average KS after using the mobile app (African American KS = 29.8(3.9), range= 14.0-34.0; Caucasian KS= 31.3(3.4), range=29.0-34.0; *P* value = .06). Thus, after using the mobile app, the disparity in the smoking knowledge score between African American and Caucasian subjects became insignificant.

**Figure 1:**
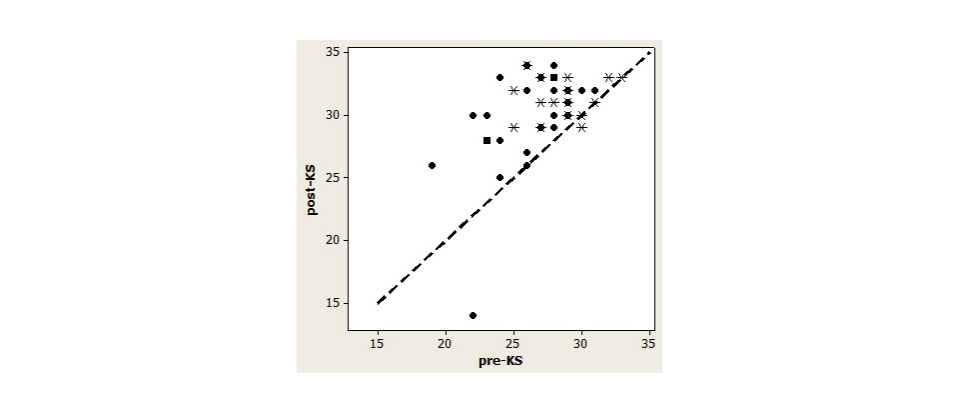
Scatterplot of post-KS against pre-KS values stratified by race (circles: African Americans, squares: American Indians/Alaska Natives, stars: Caucasians).

Figure 1 depicts a scatterplot of post-KS against pre-KS scores by race. All points above the diagonal line represent gains in knowledge. All study participants except one African American and one Caucasian participant achieved a higher KS post using the mobile app. Both of these patients were recently admitted to the unit and their condition was not fully stabilized. All patients with baseline KS < 25 were African American, and all but one achieved knowledge gains following the mobile app use.

**Figure 2:**
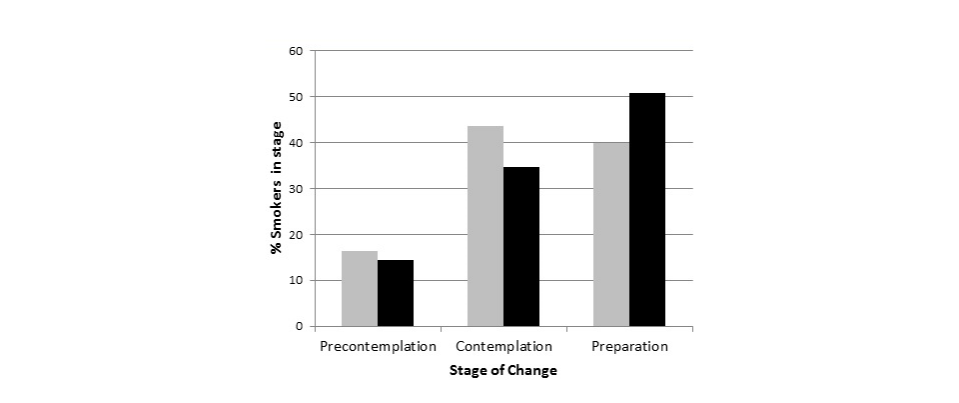
Distribution of stages of change before and after the mobile app use (gray bars: before the app use, black bars: after the app use; see detailed description in the text).

Figure 2 depicts distribution of study participants across the TTM stages of change pre- and post-mobile app use, based on the Process of Smoking Cessation survey. Overall, 13% (7/55) of patients moved toward more advanced stage of change. The percentage of participants in the precontemplation and contemplation stages decreased after mobile app use with a corresponding 11% increase in percentage of patients reaching the preparation stages. Proportion of patients in precontemplation stage decreased from 16% (9/55) to 14% (8/55) whereas the proportion of patients in the preparation stage increased from 40% (22/55) to 51% (28/55).

The mobile app positively affected patient attitudes regarding smoking cessation (Table 3). At the baseline, 36% (20/55) of patients felt they “cannot quit smoking.” After computer-assisted education, the proportion of patients who felt they “cannot quit smoking” reduced to 18% (10/55). Two-sided chi-square test showed that this change was statistically significant (*P*<.03). Other attitudes related to desire to quit smoking, feeling a loss after quitting smoking, believing that risks of smoking are exaggerated, and assessing risks of dying from smoking demonstrated improvements in a positive direction (Table 4). The Smoking Self-Efficacy Survey showed a decrease in mean scores from the pretest to the posttest demonstrating perceived reduction in temptation to smoke (Table 3) after the tablet use; however, this change did not reach statistical significance. The Decisional Balance Scale showed modest improvements which did not reach statistical significance.

**Table 4:** Attitudinal survey (N=55)

	Option^a^(%)			
Question	1	2	3	4
1. How complicated was it to use the computer?	2	4	6	88
2. Did you have any difficulty moving from one screen to another?	90	10	0	0
3. How difficult was it to use the keyboard/mouse?	2	0	10	88
4. Did you have any difficulty reading text from the computer screen?	94	6	0	0
5. Was the size of the text presented on the screen sufficient?	94	4	0	2
6. Did you like the colors used on the computer screen?	82	14	4	0
7. Did you like the audio/visual content provided by the computer?	82	14	4	0
8. Did you get all the necessary information about using the computer during initial practice session?	88	12	0	0
9. Did you come across any unknown words which were not explained by the computer?	4	4	14	78
10. How difficult were the sentences used in the educational materials?	2	2	14	82
11. How much new information did you get using the computer?	47	41	8	4
12. Did you get any feedback from the computer about your learning progress?	59	31	8	2
13. How frequently did you find the information confusing?	6	10	37	47
14. How frequently did you find educational contents difficult to understand?	2	12	20	65
15. Did you have to wait for new information to come up on the screen?	2	6	14	78
16. Would you like to use this type of computer education in the future?	76	23	0	2
17. Would you advise other patients to use computer education?	92	6	2	0
18. Overall how would you grade this learning experience?	0	8	16	76

^a^The following options were used for the questions above (in the ascending order): #1: Very complicated, Moderately complicated, Slightly complicated, Not complicated at all #2, #4: Not at all, Very rarely, Frequently, All the time #3, #10: Very difficult, Moderately difficult, Slightly difficult, Not difficult at all #5: Fully sufficient, Sufficient almost all the time, Sufficient some of the time, Not sufficient at all #6, #7: Certainly yes, To a large extent, To some extent, No #8: All information, Almost all information, Partial information, Very limited information #9: Very significant amount, Considerable, A few, None #11: Very significant amount, Considerable, Little, Very little #12, #15: All the time, Occasionally, Very rarely, Never #13, #14: Very frequently, Occasionally, Very rarely, Never #16, #17: Certainly yes, Maybe, Unlikely, No #18: Needs serious improvement, Satisfactory, Good, Excellent

### Factors Affecting Users’ Knowledge Gain

To ascertain what factors affected successful improvement in hazards of smoking knowledge after using the mobile app, a multivariate linear regression analysis was performed with pre/post DKS as dependent variable, and baseline KS, age, gender, race, education level, working status, app acceptance, and computer skills level as independent covariates. The analysis demonstrated significant relationship between baseline KS and subsequent knowledge gain after using the mobile app, with lower baseline knowledge scores predicting a higher knowledge gain after using the app (*P*<.02). Other significant factors affecting knowledge gain were working status and app acceptance. Being fully employed (*P*<.05) and having high acceptance of the app (*P*<.01) were associated with higher knowledge gain from using the app. No significant influence on knowledge gain was found for other independent variables including age, gender, race, educational status, and computer skills.

### Factors Affecting Patient Acceptance of the Mobile App in a Hospital Setting

Table 4 details results from the attitudinal survey administered to each participant after their use of the mobile app. Overall scores on attitudinal survey ranged from 53 to 72 with mean score of 67(4). Eighty-eight percent (48/55) of the participants reported that the mobile app was not complicated at all, 96% (53/55) liked the colors used on the screen and the audio/visual content, 88% (48/55) reported obtaining considerable amount of new information, 92% (51/55) answered that they would recommend other patients to use such an app, and 98% (54/55) of the patients were willing to use such an app in the future.

In order to identify factors affecting patient acceptance of the mHealth education app, a multivariate logistic regression analysis was performed with attitudinal survey score as the dependent variable, and age, gender, race, educational status, computer skills, income, and DKS, as independent covariates. For this analysis, attitudinal survey score and DKS were dichotomized to high/low acceptance and high/low knowledge gain levels correspondingly based on their distribution. The main factor significantly affecting acceptance of the mHealth education app was knowledge gain (DKS). People with higher knowledge gain after using the app were 4.8 times more likely to exhibit more favorable attitudes toward the mobile app (odds ratio (OR)=4.8; 95% confidence interval (CI) (1.1, 20.0)). Other covariates such as age, gender, race, income, education, and computer skills did not appear to significantly affect the mobile app acceptance by the hospitalized smokers.

During the semistructured interviews, participants were asked to share their experiences and provide suggestions in three areas related to the mobile app: content, interface, and value of the program. Table 5 highlights common themes and key quotes from the semistructured interviews. The participants stated that they favored the interactive computer-assisted learning over other methods like books, magazines, videos, compact discs, or talking to health care providers while in the hospital. Ninety-six percent (53/55) said that the interface design was easy and fun to use, 88% (48/55) stated that they learned significant amount of new information, and 92% (51/55) recommended this type of computer-assisted education for other hospitalized patients. Ease of operation was high, with 85% (47/55) of the participants stating that they did not need help while using the system. Most participants mentioned that the multiple choice questions helped to reinforce the material, however, one participant commented that open-ended questions might be helpful too. Of great interest is that the majority of the participants stated that the app helped them consider quitting smoking. As one of the participant stated, "This is a good program to change people’s minds who smoke. I start thinking about quitting now. This program made me want to quit smoking."

**Table 5:** Examples of qualitative feedback on the mobile app.

Key quotes from the semi-structured interviews		
**Content**
**Information value**	**Comprehension problems**	**Improvement suggestions**
It gives information you need; I’ve learned a lot; it was very educational to me. I thought I knew it all, but I didn’t know a lot.	I feel I do not know enough, I am not good enough to understand; a couple of questions were little confusing.	I would like more of the scientific info messages like the number of poisonous elements in a cigarette; include more scary disease related subjects, to show – this is what you are doing to yourself; simpler words, smaller sentences; add video clips; a little animated character like GEICO lizard; more animation would be better; make it into the video game. A super hero stops people on streets and saves them from smoking. Crossword puzzle – all bad words about smoking cigarettes.
**Interface**			
**Problems**	**Ease of use**	**Improvement suggestions**
		
There was slight pause (delay) between the message and the quiz. It made me skip to the quiz directly; sometimes I had to click couple of times to move to next screen; I hit a wrong button once but I caught up.	I didn’t have any difficulty; just click.	Something other than clapping: for example, Ta-da sound I would make it simple. Simple is the best; may be male voice; may be music on the background; bigger screen; headphones; I would like to be able to go back to the content (message) if I am not sure. Addition of ‘Back’ and ‘Forward’ button would be helpful.
**Program process**		
**Attitudes toward computer**	**Program usefulness**	**Program effectiveness**
		
I don’t like flipping pages. Paper is cumbersome. Computer is self-contained. I have more control with computer. Quicker and better with computer than learning from brochures; I like Hands On, an interactive part, the computer was better – I would NOT read hand out brochure; computer is much better – you got to see images. Radio is just read, TV got images, but here you have it all.	I think people need to do this program to learn about smoking. I think more people need to do this program to learn what they are breathing into their bodies; kids are using computers. Getting to kids through computer will get the message of hazards of smoking across to them. Get it to schools.	I am going to quit now. I will not pick up a cigarette ever in my life; This is a good program to change people’s minds who smoke. I start thinking about quitting now. This program made me want to quit smoking.

## Discussion

### Principal Findings

In this study we demonstrated that delivery of a 45-minute interactive education via a mobile app on hazards of smoking for hospitalized smokers is feasible and associated with a statistically significant increase in hazards of smoking knowledge levels as well as positive changes in patient smoking attitudes, self-efficacy, and readiness for quitting based on stages of the TTM. These positive effects were demonstrated regardless of gender, race, educational level, or computer skills. Significant determinants of successful use and acceptance of the mobile app by hospitalized users were identified.

The study provided important insight on sociobehavioral characteristics of hospitalized smokers understanding of which is essential in developing mobile apps for hospitalized smokers. Majority of them had a long history of tobacco consumption with FTND scores indicating high levels of nicotine dependence. Despite a smoking ban at the hospital premises, 20% (11/55) of the participants indicated that they smoked during their hospital stay. Over three-quarters of these patients were hospitalized for emergency treatment with alcohol and drug abuse being the most frequent comorbid condition (20/55, 36%), followed by depression or other emotional problems (19/55, 34%). Due to high level of distress at admission, the patients were offered mobile app only at the second or third day of their hospital stay. The mean number of smokers in the household of hospitalized smokers was 2.0(1.5). Thus, education about hazards of secondary smoking as well as involvement of household members in a smoking cessation program is necessary for these patients. Despite high levels of nicotine addiction, over 80% of the patients tried to stop smoking in the past and majority stated that they would like to give up smoking in the future. The distribution of stages of change in the hospitalized smokers and in general population differed remarkably. In our study initial distribution of smokers in precontemplation, contemplation and preparation stages was 16%, 44%, and 40% whereas in general population this distribution was reported to be 37%, 47%, and 16%, correspondingly [61]. High proportion of people in contemplation and preparation among hospitalized smokers coupled with inability to achieve maintenance in smoking cessation in the past underscores high importance of assistance in smoking cessation that should be provided to these patients during their hospital stays. Given that the mobile app positively affected cognitive and behavioral factors associated with smoking cessation, it can be used as an integral component of a hospital-based smoking cessation program.

Most of our study participants were African Americans, were unemployed, and had low-education and low-income levels. The majority of patients had very limited computer education. At baseline, Caucasians, employed patients, and those with higher-education level demonstrated better knowledge about hazards of smoking but all groups appeared to benefit from this intervention regardless of their background. Differences in baseline knowledge were significant between African Americans and Caucasians; however, after using the mobile app the knowledge scores in both groups increased, and the difference in the mean KS between these two groups became insignificant. Thus, the mobile app helped to decrease racial disparity in health literacy as it pertains to the hazards of smoking knowledge.

The mobile app used in this study aimed to increase patients’ knowledge levels about hazards of smoking and was successful in doing that regardless of race, gender, computer skills, or educational level. The mobile app delivered a very simple interactive curriculum that can meet the educational needs of patients who are able to read at a fifth grade level. It is likely that the consistently positive impact of the mobile app on knowledge gains among the study participants is related to the app’s interface features, such as specifically developed content for low literacy users, one message per screen, question and answer format, voice-over option to address functional illiteracy, and use of illustrations as content anchors. Based on the regression analysis, the main predictor of knowledge gain was the baseline knowledge score. This is anticipated because with higher baseline scores, there is not as much room for knowledge gains resulting in a ceiling effect.

In regard to hospitalized smokers’ readiness for change, our results are consistent with earlier studies showing that the majority of hospitalized smokers are in the contemplation and preparation stages. In a recent study, among hospitalized patients, Katz et al [62] reported that 61% of their study participants were ready to quit immediately; another study among hospitalized patients with cardiac diagnosis showed that three quarters were thinking of quitting within 6 months with approximately half of those ready to set a quit date [13]. In our study, participants were admitted for a variety of reasons, often less life threatening than an acute cardiac condition and many unrelated to smoking. Therefore, it is not surprising that our patients were less prepared to quit with 40% (22/55) in the preparation stage. After using the interactive education app, an additional 11% (6/55) of patients reported that they were ready to quit. This is consistent with earlier studies showing that even brief interventions can have a significant impact on smokers’ attitudes [9,11,63]. This is particularly relevant given that extensive hospital-based interventions, though effective in increasing quit rates, have been difficult to translate into practice until recently [9,12]. Given the current health care climate, interventions that are not resource intensive and can reach a large number of smokers are needed, and these interventions should be designed to easily fit into the hospital workflow.

Participants’ attitudes toward the mHealth education app in hospital were largely positive. Those who had more knowledge gains scored better on the attitudinal surveys suggesting that they perceived more value and benefits from the mobile app use. The majority of participants reported learning new information from using the mobile app, and more than 90% (51/55) of the participants reported that they would certainly recommend it to other smokers. It is conceivable that additional similarly designed modules incorporating smoking cessation counseling features, in addition to education might provide a feasible and effective approach to deliver smoking cessation counseling to large numbers of hospitalized smokers. Nevertheless, the impact on smoking cessation rates might still be modest in absence of outpatient follow-up [64-65]; whether these outpatient interventions might be facilitated by a mobile app used in a hospital remains to be determined. In a recent Cochrane review of inpatient smoking cessation programs, inpatient interventions that involved intensive counseling and continued for at least 1 month post discharge were associated with increased smoking cessation rates (OR=1.65; 95% CI (1.44,1.90)) [10,66]. If interventions based on mobile apps prove to be similarly successful, they could result in a major public health impact. More research is needed to develop and evaluate such interventions, to test their large-scale feasibility, and to extend their use beyond the hospital.

### Strengths and Limitations of the Study

A strength of this study includes the demonstration of high acceptance of the mobile app by hospitalized smokers who are in greatest need of smoking cessation intervention including minority patients with low socioeconomic status and limited education [67,68]. Despite these potential barriers, and the limited exposure to computers, the patients in this study found the mobile app usable and educational. Limitations of this study are the relatively small sample size, quasi-experimental design, and short follow-up. Specifically, although patients reported a higher readiness to quit smoking following the mobile app intervention, we did not determine whether these patients ultimately maintained a desire to quit following hospital discharge, and we do not know how many of them were successful in doing so.

### Implications for Future Research

Statistically significant knowledge gain achieved by the mobile app users described in this study concurs with our previous studies [31-35]. As in the previous studies, knowledge gain coincided with improvements in attitudes and beliefs [50]. For example, computer-assisted depression education resulted not only in improvement in depression knowledge but also in decrease of mental health stigma [69]. In this study, besides increase in hazards of smoking knowledge, statistically significant improvements in self-efficacy and smoking attitudes were achieved. Also positive shifts in stages of change and decisional balance were identified, though they didn't reach statistical significance. This may be explained by a relatively small sample size and heterogeneity of study sample in terms of patients' background and smoking behavior. In addition, because hospitalized smokers included high proportion of individuals with high levels of nicotine dependence coupled with low socioeconomic status and limited education, more intensive and multicomponent interventions over prolonged period of time may be warranted to achieve sustainable change in smoking attitudes and behaviors leading to smoking cessation and lasting smoking abstinence. Thus, lack of significant change in Decisional Balance Scale in our study may be attributed to the fact that changing Pros and Cons balance in habitual smokers requires multifaceted intervention beyond a single encounter with a mobile app.

Multiple studies demonstrated that knowledge and beliefs about smoking are associated with key behaviors such as cessation and intent to quit. [70,71]. A consistent relationship between smoking status and belief in the harmfulness of smoking is well described [72]. Previous studies showed that those who evaluate smoking behavior negatively do so at least in part because they have knowledge of the negative health effects of smoking and this negative evaluation contributes to the intention to not smoke [73]. Our study concurred with these reports by showing various gaps in hazards of smoking knowledge in hospitalized smokers particularly on association of tobacco use and increased risk for leukemia, colon cancer, and cervical cancer. Knowledge gains attained after the mobile app use resulted in positive shifts of smoking attitudes and other behavioral constructs affecting quitting intentions. However, increasing knowledge about the harmful effects of smoking may not be sufficient for smoking behavior change and other factors such as social norms may play significant role [74]. Knowledge gain achieved by health education is generally considered a prerequisite for a successful behavior change and it may be helpful in affecting attitudes and beliefs however it is usually not sufficient to achieve lasting behavior change [53-56]. Different behavior change theories describe various constructs affecting health behaviors. For example, Theory of Planned Behavior (TPB) describes health behaviors and intentions including smoking using such constructs as attitudes, subjective norms, and perceived behavioral control [75]. Health education may be instrumental in affecting some of these constructs but additional intervention components are needed for successful behavior change. Based on underlying behavioral constructs emanating from corresponding behavior change theories such as TTM or TPB, effective mobile app for smoking cessation should be able not only educate but provide tailored counseling that corresponds to individual psychological, behavioral, and social characteristics of a smoker over a prolonged period of time. Interactive educational components aimed at increasing knowledge and tailored counseling components aimed at changing behaviors play complementary roles in mobile apps for smoking cessation. Thus, the mobile app described in this study may be a part of a multifaceted smoking cessation intervention delivered over a prolonged period of time in a tailored personalized way via multiple health communication channels as it was described previously [40].

Our study results concur with previous reports on positive use of hospital-based patient education [76,77]. Though earlier studies acknowledged significant potential of hospital settings as a fruitful venue for engaging patients in their care and empowering them with individualized health education and counseling, limited resources and personnel shortage were frequently identified as barriers toward widespread implementation of hospital-based patient education [76,77]. Previous studies reported that during hospital stays many patients experienced substantial inactive time coupled with recognition of seriousness of their health condition and desire to learn more about their care; however, staff availability for personalized health education was limited [78]. Our study demonstrated that mobile apps for personalized health education may help successfully address this barrier. Recently published studies confirm high potential of mobile apps delivered via tablet computers or smartphones for patient-centered hospital-based programs aimed at patient education, engagement, and empowerment [78-80]. Growing number of clinicians endorse use of tablets for personalized care delivery to their patients [81]. Employing more comprehensive computer-assisted interventions using a variety of behavioral constructs tailored to individual patient profiles over prolonged period of time may significantly enhance the performance of such systems in the future [79]. Larger scale studies with extended follow-up will be required to definitely evaluate the clinical benefit of this type of intervention [78].

### Conclusions

A mobile app provides feasible and effective means to educate patients about the hazards of smoking in a hospital setting. The mobile app has significant potential in facilitating the reduction of racial disparities in health literacy as it pertains to hazards of smoking knowledge. Further research is needed to evaluate the cost-effectiveness and long-term effects of this promising patient engagement and empowerment approach.

## Abbreviations

CI: confidence interval
CO-ED: COmputer-assisted EDucation
DKS: difference in knowledge score
FTND: Fagerstrom test for nicotine dependence
KS: knowledge score
OR: odds ratio
SD: standard deviation
TPM: theory of planned behavior
TTM: transtheoretical model
